# Analysis of WRKY Resistance Gene Family in *Boehmeria nivea* (L.) Gaudich: Crosstalk Mechanisms of Secondary Cell Wall Thickening and Cadmium Stress

**DOI:** 10.3389/fpls.2022.812988

**Published:** 2022-03-28

**Authors:** Xinkang Feng, Aminu Shehu Abubakar, Chunming Yu, Aiguo Zhu, Jikang Chen, Kunmei Chen, Gang Gao, Xiaofei Wang, Pan Mou, Deyi Shao, Ping Chen

**Affiliations:** ^1^Institute of Bast Fiber Crops, Chinese Academy of Agricultural Sciences, Changsha, China; ^2^Department of Agronomy, Bayero University Kano, Kano, Nigeria

**Keywords:** ramie, WRKY transcription factor, expression profile, cadmium stress, GO, KEGG, crosstalk

## Abstract

A total of 60 WRKY family genes of ramie were identified in the ramie. The genes were unevenly distributed across 14 chromosomes in the specie and highly concentrated (72%) in the distal telomeric region. Phylogenetic analysis placed these genes into seven distinct subfamilies groups: I, II (a, b, c, d, e), and III, with group IIc containing only the variant of heptapetide sequence (WRKYGKK). Segmental duplication events (41.7%) was found to be the main driver of BnGWRKY evolution. Thirty eight from among the genes showed collinear relationships with WRKY genes from *Arabidopsis thaliana*, *Cannabis sativa, Oryza sativa*, and *Zea mays*. The number and density of stress and hormone responsives *cis*-acting elements were comparably higher than other elements, with abundant ARE and rare LTR *cis*-acting elements indicating the long-standing adaptability of ramie to its natural environment. GO and KEGG enrichment analysis of the WRKY target genes revealed their involvement in response to stimuli, immune system processes, transporter protein activity and antioxidant activity. Expression analysis show that most WRKYs were activated by the cadmium stress, more especially the *BnGWRKY2*, *BnGWRKY15*, BnGWRKY20, *BnGWRKY50* and *BnGWRKY58*. Combining transcriptome, orthologous gene relationships and qPCR result, we established the possible involvement of *BnGWRKY50* and *BnGWRKY58* in crosstalk mechanism between secondary cell wall thickening and Cd^2+^ stress. This provided information into the role of BnGWRKY proteins in ramie secondary wall development and cadmium stress response to, and could serve as basis for improvement of the ramie.

## Background

In recent years, extreme natural environments have had a tremendous impact on plants, causing huge economic losses. Therefore, evolving a complex signaling system evolution is crucial for the survival and continuation of plants. Signaling events include reception, interaction and adaptive response to the environmental changes (Yoon et al., 2015), and transcription factors have been identified as crucial players in this. Interaction of specific transcription factors (transcriptional regulatory proteins) binding to corresponding cis-elements in the promoter regions of target genes is remarkable in ensuring plant survival under adverse environmental conditions. WRKY transcription factors are one of the largest families of transcriptional regulators in plants (Eulgem et al., 2000). Numerous expression and mechanistic studies have provided insights into the engagement of WRKYs in different areas of plant biology, particularly in response to abiotic and biotic stresses, and thus WRKYs have been reputed resistance family. Overexpression of three WRKYs, *GmWRKY136*, *53* and *86*, in the soybean cyst nematode (SCN) susceptible cultivar Williams 82 increased the level of SCN resistance by up to 55% (Yang et al., 2017). Another WRKY from *Capsicum annuum* (*CaWRKY27*) overexpressed in tobacco positively regulated its resistance to *Ralstonia solanacearum* infection through modulation of SA, JA, ethylene, and NO-mediated signaling pathways (Dang et al., 2014). Buckwheat *FtWRKY46* is reported to have improved tolerance to salt stress through an improved ROS scavenging system (Lv et al., 2020) and *CsWRKY46* in cucumber regulated a series of cold stress response genes in an ABA-dependent manner, thereby enhancing cold resistance in transgenic plants (Zhang et al., 2016). In a similar manner, Luo et al. overexpressed *GsWRKY20* in *Arabidopsis* and found it to have enhanced drought tolerance by regulating stomatal closure and reducing stomatal density through ABA signaling which reduced water loss (Luo et al., 2013). Studies on the involvement of WRKY in the regulation of Cd^2+^ stress have also been demonstrated in *Arabidopsis* by Han et al. (2019). They found that *WRKY12* directly targets *GSH1* and indirectly suppresses PC synthesis-related gene expression and negatively regulated Cd^2+^ accumulation and tolerance in *Arabidopsis*. In different study, Zhang et al. (2020) found Cd^2+^-induced *AtWRKY13* to have activated *DCD* and *PDR8* (as a Cd^2+^ extrusion pump) expression and consequently resulted in increased production of H2S and enhanced plant tolerance to Cd^2+^. WRKY genes also play an important role during stem development, especially during secondary cell wall thickening. *AtWRKY13* reported to partakes in stem development by binding to *AtNST2* promoter, which regulates *AtNST2* gene expression during SCW synthesis and lead to rescue of weaker stem (Li et al., 2015). *AtWRKY12* expression causes *NST2* and *C3H* zinc finger TF to be derepressed, which in turn activates the biosynthesis of xylan, cellulose and lignin required for secondary wall thickening (Wang H. et al., 2010). Ectopic expression in tobacco revealed that *VvWRKY2* activates the promoter of the *VvC4H* gene involved in the lignin biosynthesis pathway and regulates lignification in grapevine (Guillaumie et al., 2010). Potato *StWRKY1* regulates the secondary cell wall thickening gene (*HCAA*), which in turn affects the expression of *4CL* and *THT* (Wang and Dixon, 2012). Interestingly, WRKY mediates the crosstalk mechanism between abiotic and biotic stress responses. For example, *OsWRKY45* (Qiu and Yu, 2009) and *SlWRKY8* (Gao et al., 2020) were reported as positive regulators of PR gene expression, drought and salt stress tolerance as well as ABA sensitivity. *AtWRKY40* and its tomato homologs *SlWRKY39* and *SlWRKY45* increased tolerance to pathogen infection and multiple abiotic stresses (Shen et al., 2007; Sun et al., 2015; Chinnapandi et al., 2017). The defense responses involved in WRKY transcription factors are a complex network of cross-regulation (Jiang et al., 2017), and their interactions provide a basis for studying crosstalk mechanisms in different biological processes.

In recent years, WRKY families of an increasing number of species have been reported, for example, *A. thaliana* (72) (Eulgem et al., 2000), *O. sativa* (109) (Rushton et al., 2010), *Ananas comosus* (54) (Xie et al., 2018), *Linum usitatissimum* L. (102) (Yuan et al., 2021), *Cucumis sativus* L. (61) (Chen et al., 2020b), *Spirodela polyrhiza* (47) (Zhao et al., 2021), etc. WRKY TFs have a conserved DNA-binding structural domain of 60 amino acids with a highly conserved WRKYGQK sequence at the N-terminal end and a zinc finger pattern at the C-terminal. There are also several variants of the conservative WRKYGQK, such as WRKYGKK, WKKYGQK, WRKYGRK, WRKYGEK, and WSKYEQK (Zhao et al., 2021). WRKY TFs regulate target genes by recognizing and binding W-box cis-acting elements (TTGACC/T), and the number of W-boxes becomes a marker of the strength of the interaction with the target gene.

Abiotic or biotic stress resistances are very important for the survival of plants, especially perennial crops, which spends times (>3 years) growing and passes through various natural changes in the environment such as cold winters and hot dry summers, so the empirical for developing their own unique resilience mechanism in the natural long-term selection (Luan et al., 2018). Ramie is one of such perennial plants and is grown for its best quality fiber coupled with its higher heavy metal tolerance as a soil remediation crop especially in mining areas in southern China (Feng et al., 2021). This robust nature of the plant made us proposed the possible involvement of WRKY family in heavy metal such as cadmium tolerance (Zhu et al., 2020), drought tolerance (An et al., 2015), and cold tolerance. We conducted a genome-wide study of the resistance family WRKY to elucidate the regulatory network and mechanism of action of WRKY family genes in ramie with respect to fiber development and cadmium stress. In this study, we identified 60 ramie WRKY genes and performed a comprehensive analysis of gene structure, motif composition, chromosome distribution, collinearity analysis within and outside the species, GO and KEGG of target genes, expression pattern and protein interaction network. Several cadmium-responsive genes were initially identified and envisaged a possible crosstalk mechanism, which provided valuable information into functional identification of ramie WRKY gene family members.

## Results

### Identification and Analysis of BnGWRKY Genes

Using the 72 *Arabidopsis* WRKY as query, we identified 60 *WRKY* genes in ramie based on the presence of apparently complete WRKY domains and renamed *BnGWRKY1* to *BnGWRKY60* based on their order on the linkage groups (Supplementary Table 1). Chr position, molecular weight (MW), isoelectric point (pI), conserved motif, zinc finger domain pattern, subcellular localization prediction result, gene nucleotides, and protein were also computed and presented also in Supplementary Table 1. Among the 60 BnGWRKY proteins, *BnGWRKY21* was the smallest protein with 136 amino acids (aa), and *BnGWRKY11*(738 aa) was the largest with a molecular weight of 15.5 and 80.0 kDa respectively. The isoelectric point (pI) ranged from 4.85 (*BnGWRKY17*) to 10.02 (*BnGWRKY22*). All the BnGWRKY are predictably localized in the nuclear region.

### Multiple Sequence Alignment, Phylogenetic Relationship, and Classification of BnGWRKY Proteins

**FIGURE 1:**
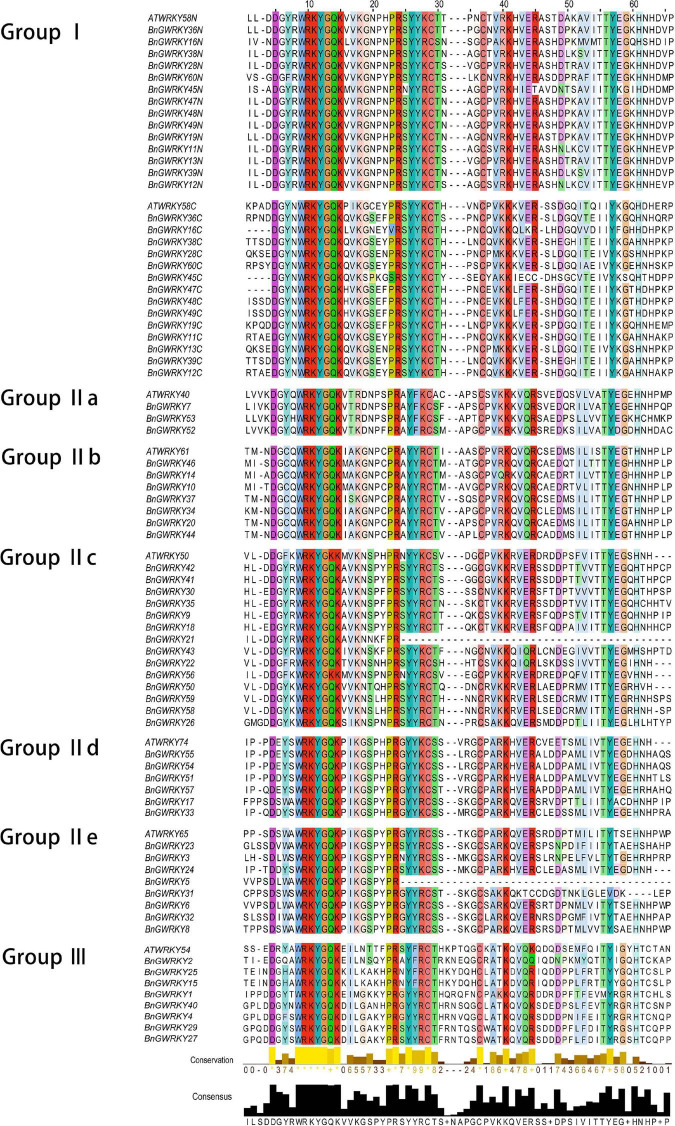
Multiple sequence alignment of the WRKY domains of BnGWRKY and selected AtWRKY domain. “N” and “C” indicate the N-terminal and C-terminal WRKY domain of a specific WRKY protein.

**FIGURE 2:**
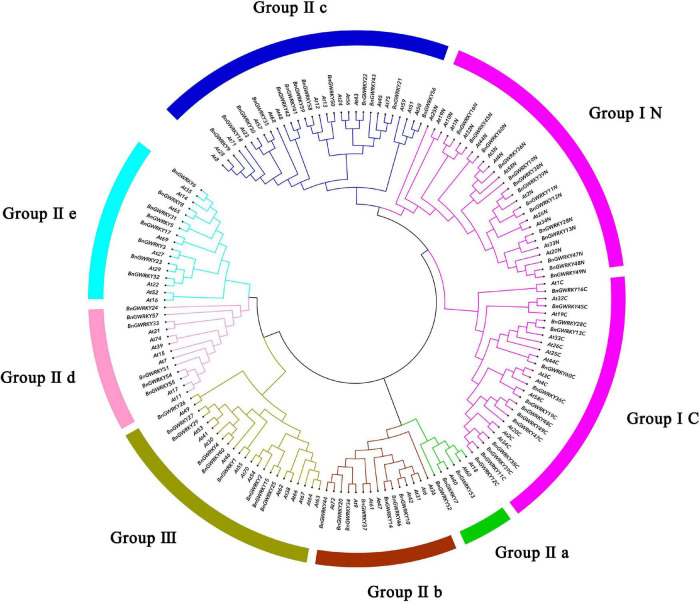
Phylogenetic tree (Guo et al., 2019) representing relationships among WRKY domains of *Boehmeria nivea* and *Arabidopsis thaliana* using MEGA-X with 1,000 bootstrap replicates. The arcs with different color represent seven subgroups of WRKY proteins. Group I proteins with the suffix “N” or “C” indicates the N-terminal or the C-terminal WRKY domains.

Multiple sequence alignment of the 60 BnGWRKY proteins and seven AtWRKY is shown in Figure 1. The highly conserved WRKYGQK sequences were the dominant with BnGWRKY56 (WRKYGKK) occurring in group IIc being the only exception. According to the constructed phylogenetic tree of the BnGWRKYs and AtWRKYs (72), the BnGWRKYs could be divided into three large groups corresponding to group I, II, and III in *Arabidopsis* as defined by Eulgem et al. (2000) (Supplementary Figure 1). There were 14 BnGWRKYs in Group I, 38 in Group II, and 8 BnGWRKYs in Group III. This is consistent with unrooted phylogenetic tree constructed (Figure 2) which revealed all the 14 members from Group I to have contained two WRKY domains (an N-terminal and a C-terminal WRKY domain) and also harbored distinct C2H2-type (C-X4-C-X22-23-H-X-H) zinc-finger (Supplementary Table 1). Members of Group II which are unequally distributed among the five subgroups: II a (*n* = 3), II b (*n* = 7), II c (*n* = 14), II d (*n* = 6), and II e (*n* = 8) however contained a single WRKY domain. Although most of the group II members had integral C2H2-type zinc finger motifs, the motif is absent in *BnGWRKY5* and *BnGWRKY21* (Figure 1). The eight BnGWRKYs members in Group III harbored WRKYGQK sequence and C2HC-type zinc-finger (Figure 1).

### Gene Structure and Motif Composition of BnGWRKYs

**FIGURE 3:**
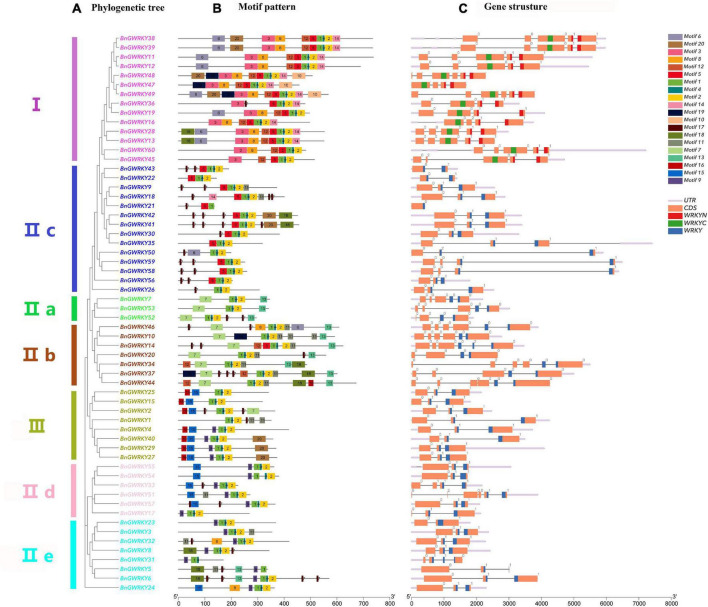
Phylogenetic relationships, conserved motifs and gene structure of WRKY genes from ramie. **(A)** The neighbor-joining tree based on the full-length protein sequences of 60 *Boehmeria nivea* WRKYs with 1,000 bootstrap replicates. **(B)** The motif composition of ramie WRKY proteins. The sequence information for each motif is provided in Supplementary Table 2. **(C)** The intron–exon structures of BnGWRKY genes. The WRKY domains are highlighted by blue/red/green boxes. The number indicates the phases of corresponding introns.

Gene structure of BnGWRKY family members was obtained to get additional information on their evolutionary and exon-intron structures (Figure 3A). All the genes coding sequences were disrupted by introns, except for *BnGWRKY21*. The number of introns ranges from 0 to 5 (Figure 3C). The exon–intron distribution patterns showed some similarities in number and position within the same group, especially Group III where all genes have two introns, one of which separated the conserved WRKY domain. Interestingly, the majority of intron insertions occur in WRKY conserved domains indicating the important role of these conserved domains in plants.

The result of putative motifs of the BnGWRKY proteins is presented in Figure 3B. Motifs 1 and 2 were WRKY domains and widely distributed across all the groups. All other motifs were more specific to a group (Supplementary Table 2). For example, motif 3 and motif 10 were unique to only group I, whereas motif 13 was peculiar to only Groups IIa and IIb. Similar gene structure and common motif compositions strongly support the reliability of the group classifications, further suggesting that BnGWRKYs within the same group may play similar functional roles.

### Chromosomal Distribution and Synteny Analysis of BnGWRKY Genes

**FIGURE 4:**
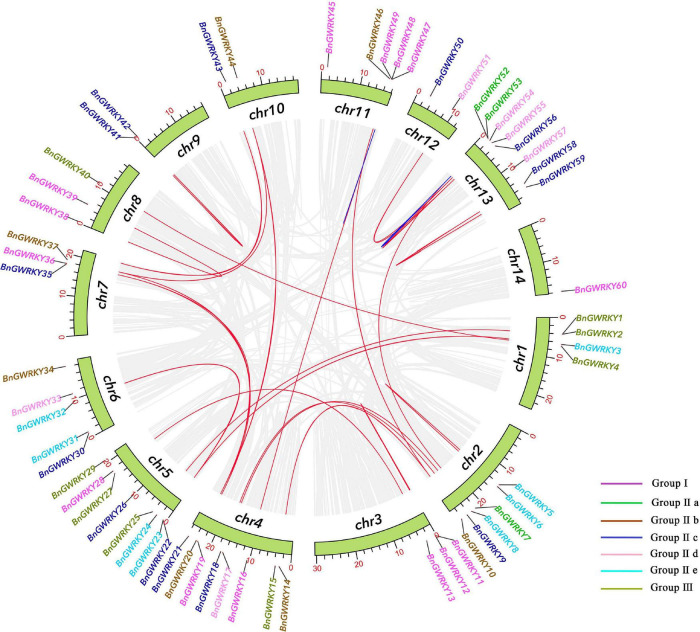
Schematic representations for the chromosomal distribution and inter-chromosomal relationships of ramie WRKY genes. Gray lines indicate all synteny blocks in the ramie genome, duplicated WRKY gene pairs are connected with red lines, blue lines indicate tandemly duplicated gene pairs. As the legend shows, the color represents the grouping of WRKY.

All identified BnGWRKYs were mapped on to 14 *B. nivea* chromosomes (Figure 4). The 60 BnGWRKYs were unevenly distributed across the 14 chromosomes with the largest number found on the 4, 5, and 13th (eight genes each; ∼13%) and the least (one genes; ∼1.7%) on the 14th chromosomes. About 72% of BnGWRKY genes were located in the telomere region. WRKY genes that belonged to the same group were scattered in all chromosomes. Gene duplication events are also shown in Figure 4 with the blue and red indicating segmental and tandem duplications respectively. There were 25 (41.7%) segmental duplication pairs between BnGRWRKY genes. Two pairs of tandemly duplicated genes were also obtained on chr11 and chr13, pointing that the WRKY genes were mainly derived from whole genome duplication events. Some of the gene pairs involved in the duplications were very close together, such as *BnGWRKY5/6, BnGWRKY11/12, BnGWRKY41/42, BnGWRKY58/59*. These results suggest that gene replication produced some BnGWRKY genes and that segmental duplication events are the major driving force in BnGWRKY evolution.

The positive pressure criteria were selected based on Lynch and Conery (2000): Ka/Ks << 1 stands for purifying selection, Ka/Ks ≈ 1 means neutral selection, while Ka/Ks >> 1 signifies positive selection. All the segmental and tandem duplicated BnGWRKY gene pairs had Ka/Ks < 1 with the exception of *BnGWRKY47/48* (Ka/Ks = 1.40) and *BnGWRKY47/49* (Ka/Ks = 1.40). Due to the occurrence of a large number of synonymous mutations, 5 segmental gene pairs were highly differentiated and have a long evolutionary distance (Supplementary Table 3).

**FIGURE 5:**
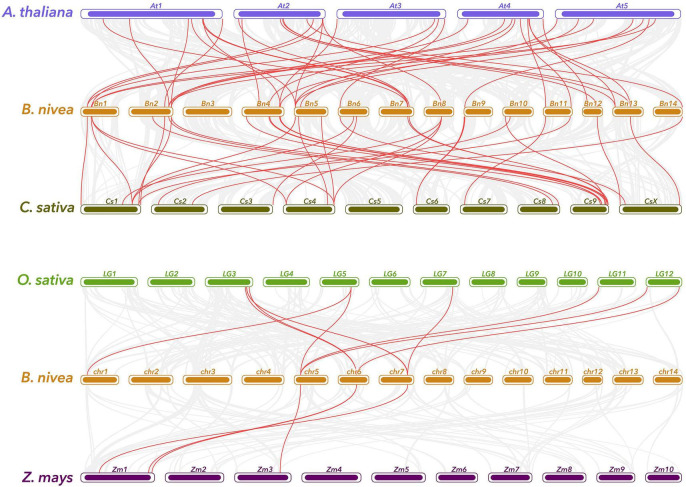
Gene duplication and synteny analysis of WRKY genes between ramie and four representative plant species. Gray lines in the background indicates the collinear blocks within ramie and other plant genomes, while the red lines highlight the syntenic WRKY gene pairs. The specie names with the prefixes “*B. nivea*,” “*A. thaliana*,” “*C. sativa*,” “*O. sativa*,” and “*Z. mays*” indicate *Boehmeria nivea*, *Arabidopsis thaliana*, *Cannabis sativa*, *Oryza sativa*, and *Zea mays*, respectively.

Synteny relationship between ramie and representatives plant species, including two dicots (*A. thaliana* and *C. sativa*) and two monocots (*O. sativa* and *Z. mays*) was also established (Figure 5). A total of 38 ramie genes have collinear relationships with 27 *A. thaliana*, 32 *C. sativa*, 5 *O. sativa*, and 4 *Z. mays* genes (Supplementary Table 4). The number of orthologous pairs between ramie and dicots were 39/37, which is far more than the number between ramie and monocots (10/4). It is worth noting that three individual ramie WRKY genes have collinear relationships with three respective genes in *Arabidopsis*. These conserved genes might share important functions across the species. Interestingly, many collinear gene pairs identified between ramie and dicots were not found between ramie and monocots indicating that these orthologous pairs formed after the divergence of dicotyledonous and monocotyledonous plants.

### Analysis of the *Cis*-Acting Elements

**FIGURE 6:**
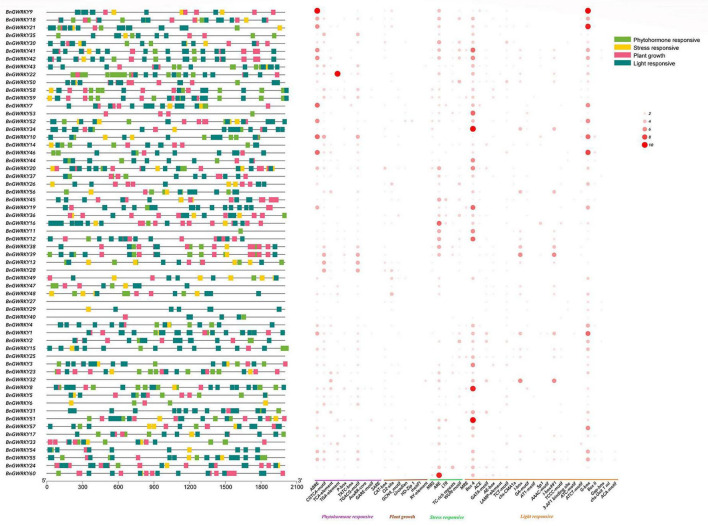
Analysis of the *cis*-elements in the promoter region of the BnGWRKY genes. The left figure shows the distribution of the upstream promoter regions (2,000 bp) of the four *cis*-acting elements. The heat map of cis-elements for phytohormone response, stress response, plant growth, light response is shown on the right, with the size of the circles indicating the number of *cis*-elements.

The promoter analysis of BnGWRKY cis-acting regulatory elements investigated the evolution and functional diversification of the WRKY. From the result, 41 *cis*-acting regulatory elements were detected, 21 from which were light responsive, nine phytohormone, six plant growth, and five abiotic stress elements (Figure 6 and Supplementary Table 5). This showed light responsive and plant growth responsive elements to be the most abundant. Gliadin metabolic response (O2-site), ARE (anaerobic induction element), Box-4, G-box (light responsiveness), and ABRE (abscisic acid responsiveness) were the most prominent elements in a separate category and more than 60 were found in the BnGWRKY genes, suggesting that these elements might play an important role in regulating gene expression. Many WRKY genes contain all types of stress responsive elements, indicating coordinated functions of WRKY gene in stress control.

### Prediction and Functional Enrichment Analysis of Potential BnGWRKY Target Genes

**FIGURE 7:**
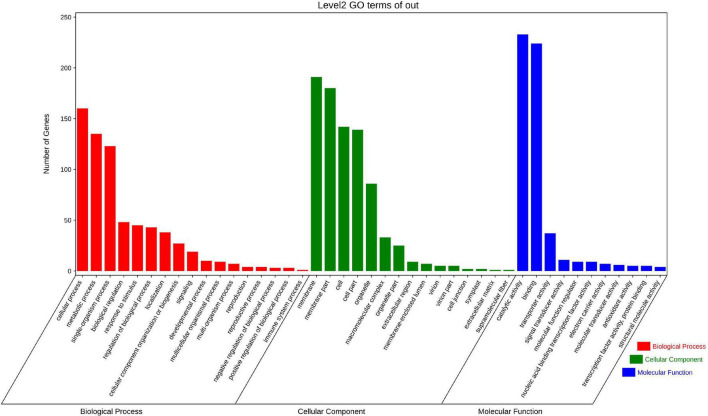
Gene Ontology (GO) analysis of WRKY target genes in *Boehmeria nivea*. Categories pertaining to cellular components, molecular functions, and biological processes were defined by GO classification.

A total of 18,950 genes having at least one W-box (TTGACC/T) in their putative promoters were identified from the assembled *B. nivea* genome. Among these number 10,290 genes have only a single W-box, 5,617 genes contained two, 2,136 genes contained three and 907 have four or more W-boxes. The 907 having at least four W-boxes were consequently were selected (Supplementary Table 6) for further functional annotation and pathway enrichment analysis. Some target genes were enriched into the GO term with respect to responses to stimulus, immune system process, transporter activity and antioxidant activity (Figure 7). The directed acyclic graph for the enriched terms based on top-GO showed viral envelope (GO:0036338, GO:0019031), toxin catabolic (GO:0009407, GO:0009404), secretion and exocytosis (GO:0046903, GO:0032940, GO:0016192, GO:0006887) (Supplementary Figure 2) revealing that WRKY transcription factors widely involved in regulation of stress.

**FIGURE 8:**
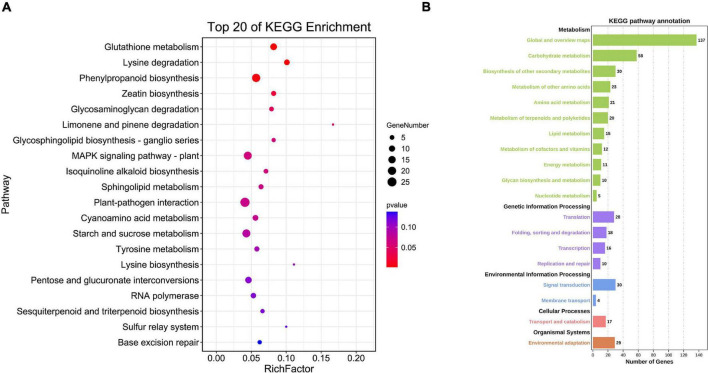
KEGG enrichment analysis of BnGWRKY potential target genes in *Boehmeria nivea*. **(A)** Top20 of KEGG enrichment. **(B)** KEGG pathway annotation.

The top enriched KEGG pathways included glutathione metabolism, MAPK signaling pathway (Chun, 2000), plant-pathogen interaction, sulfur relay system and base excision repair. Many target genes were enriched in the KEGG pathway of environmental adaptation, transport and catabolism, environmental information processing and various metabolisms (Figure 8) further indicating involvement of BnGWRKY genes in both biotic and abiotic stresses responses and other biological pathways.

### Digital Expression Patterns (RNA-Seq) of BnGWRKY Genes in Different Tissues

**FIGURE 9:**
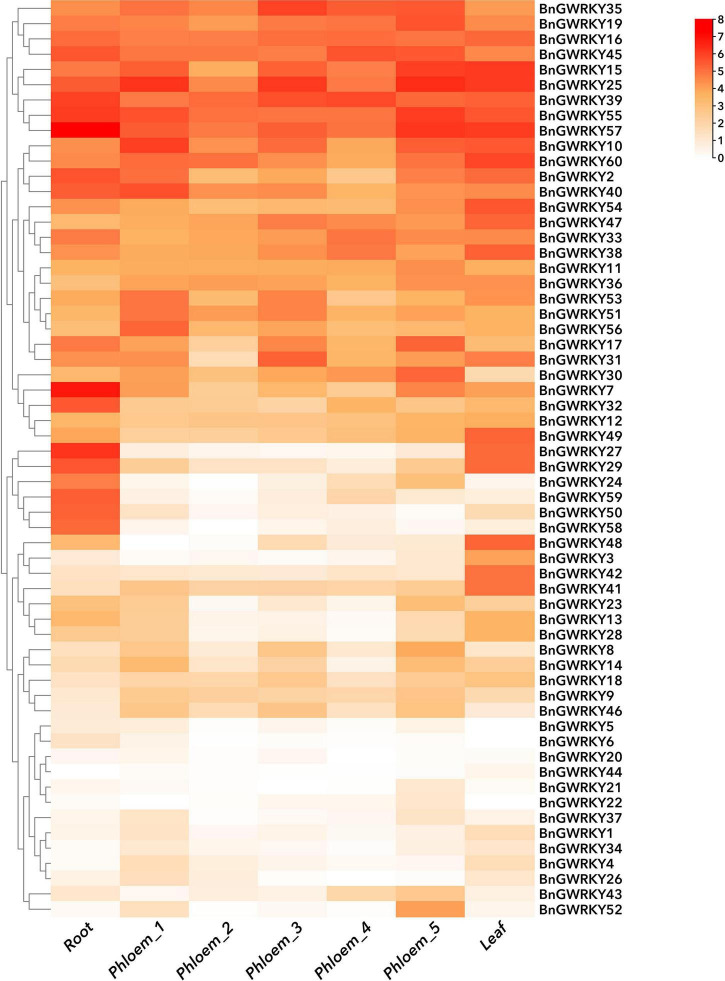
Hierarchical clustering of expression profiles of ramie WRKY genes in nine samples including different tissues and developmental stages. Log2 (FPKM + 1) values were displayed according to the color code. Detailed FPKM values were listed in Supplementary Table 7a.

A gene expression pattern may reflect its physiological regulatory function (Zhao et al., 2021). Patterns and expression levels of all the 60 BnGWRKY genes in different tissues (root, stem, leaf) and potential WRKY transcription factors involved in ramie fiber growth were studied based on the publicly available transcriptome data (Figure 9 and Supplementary Table 7a). All the genes were relatively expressed in all the tissues, pointing to the ubiquitousness of the WRKY genes (Figure 9). We performed hierarchical cluster analysis using expression data from seven other samples. BnGWRKYs were categorized into three main groups, high expression, preferential expression, relatively lower expression. We found that the phylogenetic grouping was not in agreement with the expression grouping in most cases, indicating that the expression patterns (period, location) of genes with similar functions were significantly different. Fifty-one genes were expressed in all tissues (FPKM > 0) and 10 from among showed constitutive expression (FPKM > 20). Two terms were used as screening criteria for genes with preferred expression: Tissues with the highest expression levels should have twice as much expression in at least one of the other tissues; FPKM > 2 (at least in one tissue). Fifteen genes in terrestrial root, seven genes in phloem_third period, 18 genes in leaf were found to have exhibited preferential expression over all others (Supplementary Table 7b). It is noteworthy that WRKY50 and WRKY58 have similar expression patterns, and their expression in stems and leaves were clearly lower than that in roots, which perhaps may be due to the specificity of tissue function. Nevertheless, the expressions of WRKY20, WRKY44 were low in all tissues indicating that they may require certain specific developmental stages and environments to be induced. This analysis gives us a general understanding of the expression patterns of members of this family.

### Expression Profiles of BnGWRKY Under Cd^+2^ Stress

**FIGURE 10:**
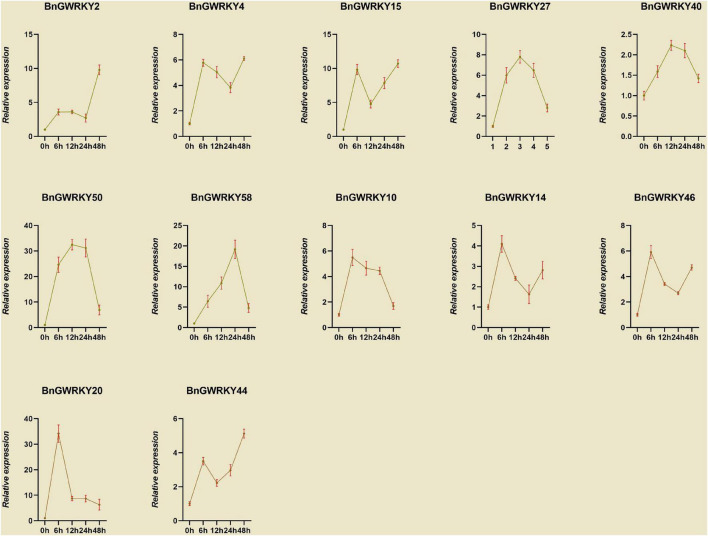
The relative expression levels of selected WRKY genes in ramie leaf at different periods under Cd^+2^ treatment (0, 6, 12, 24, and 48 h). The error bar represents the standard deviation of the three biological duplicates.

**FIGURE 11:**
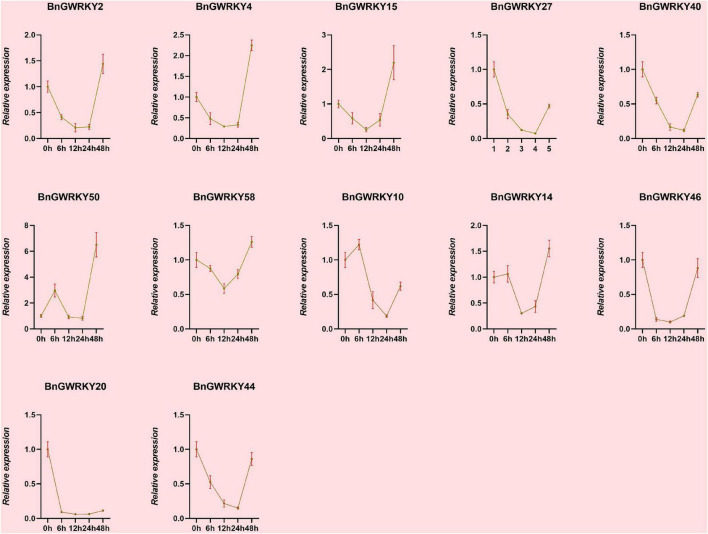
The relative expression levels of selected WRKY genes in ramie root at different periods under Cd^+2^ treatment (0, 6, 12, 24, 48 h). The error bar represents the standard deviation of the three biological duplicates.

Based on the established data of WRKYs involved Cd^2+^ stress identifies in other crops as well as transcriptome expression data, we initially hypothesized that genes in Group IIb, Group IIc, and Group III subgroups might be involved in Cd^2+^ stress response. We thus screened 12 BnGWRKY genes based on closed homologies to the identified WRKY genes from other species and conducted q-PCR analysis to investigate their expression patterns under Cd^2+^ stress. The result showed significant induction of all the BnGWRKY genes in leaves (Figure 10). *BnGWRKY2*, *BnGWRKY15*, *BnGWRKY50*, *BnGWRKY58*, and *BnGWRKY20* were induced more than 10-fold. All the induced genes showed multiple expression patterns, such as continuous rise, rise then fall, rise-fall-rise, etc., which might be related different cadmium tolerance mechanisms. Interestingly, expression of these genes was significantly suppressed in roots in contrast to leaves with most of the genes showed a decreased pattern before increasing, and only a few were highly expressed with reference to the control (Figure 11). This result may explain the different mechanisms by which different parts of the plant respond to stress. It also indicated that leaves rather than roots are the main functional organs involved.

## Discussion

### Characteristics and Evolution Pattern of WRKYs

The evolutionary tree constructed with the model *A. thaliana*, placed the 60 BnGWRKY proteins into five groups: I, IIa, IIb, IIc, IId, IIe, and III. *BnGWRKY56* protein in Group IIc, is similar to its orthologous gene *ATWRKY50*, with each having WRKYGKK instead of WRKYGQK present in all others. The conserved WRKY structural domain and zinc finger motif bonded preferentially to target genes containing W-box cis-acting elements, and variations in the WRKYGQK motif may affect the normal interaction of this transcription factor with downstream target genes, so the function and binding specificity of the *BnGWRKY56* protein deserve further investigation (de Pater et al., 1996; Zhou et al., 2008). *BnGWRKY5* and *BnGWRKY21* lack the zinc finger motif sequence which may influence its cis-element binding ability and by extension their general function. All WRKY proteins of group I in ramie have two WRKY structural domains whereas only one was present in members of group II and III. The single WRKY structural domains of members of group II and group III were reported to be more closely related to the C-terminal structural domain of group I than to the n-terminal structural domain (Eulgem et al., 2000), yet ramie does not follow this pattern: the n-terminal WRKY structural domain of ramie was not aggregated as a monophyletic subtree but was most closely related to Group IIc, suggesting that this group of proteins has different characteristics during the evolution of ramie.

Whole genome duplication (WGD) events are one of the major factors driving the evolution and expansion of family genes, especially tandem and segmental duplication events. In ramie, only an ancient hexaploidization events were detected, and no recent WGD events were found (Wang et al., 2021). This may be the reason why there are fewer ramie BnGWRKY genes than other species. It has also been shown that the number of WRKY genes is not related to genome size, and extensive gene loss, sub-functionalization or neo-functionalization may lead to family shrinkage and expansion (Sémon and Wolfe, 2007).

### Roles of BnGWRKYs in the Regulatory Network

*Cis*-acting elements are windows for genes to sense changes in the external environment and play a key role in gene initiation, regulation, and response. The prediction of promoter elements revealed the highest number and density of stress response elements (Figure 6), followed by hormone response elements, among which signaling molecule elements such as abscisic acid, MeJA, and salicylic acid were the most prominent, sensing and transmitting signals in response to external environmental changes, and maintaining plant homeostasis through hormonal networks (Dong et al., 2015). Interestingly, the abundance of ARE elements in the ramie WRKY promoter reflects the adaptability of ramie to the growth environment, which is consistent with what was obtained in aquatic plant WRKY family genes (Zhao et al., 2021). In contrast, the low-temperature responsiveness (LTR) *cis*-acting element was present in only a few WRKY genes, which simultaneously verified the adaptation of the gene to the environment (southern China) which rarely reach freezing point.

It has been shown that W-box sequences present in the promoters of many genes are the main regions of recognition and binding of the C-terminal WRKY structural domain (Eulgem et al., 2000), and therefore the number of W-box elements correlates with the reliability of the putative WRKY target genes. The target genes are mainly involved in response to stimuli, immune system processes, transporter protein activity, and antioxidant activity. In particular, the ammonium transporter protein promoted ammonium transport under salt stress conditions, which in turn reduced ammonium toxicity caused by salt stress (Bu et al., 2019), and the nitrate transporter protein also promoted nitrate uptake in Zn^2+^ stress, which in turn increased plant accumulation of Zn^2+^ (Pan et al., 2020). In addition, KEGG enrichment analysis had shown several mechanisms that play an important role in plant resistance to adversity, such as the MAPK signaling pathway, which comprised a class of proteins in linking stimulus perception to multiple cellular and adaptive responses (Danquah et al., 2014). In plants, MAPKs are involved in signaling in response to pathogens (Berriri et al., 2012), drought, salinity, cold, trauma, ozone (Moon et al., 2003), ROS (Mittler, 2002), and hormonal stimuli (Meng and Zhang, 2013).

### Roles of BnGWRKYs in Cd^2+^ Stress

It is well-known that ramie has a strong cadmium tolerance and accumulation capacity, and its products do not enter the food chain, thus making it a promising plant for the remediation of heavy metal cadmium (She et al., 2015). However, not much information is available on the involvement of WRKY genes in cadmium tolerance response. We found from q-PCR results that *BnGWRKY46*, a member of Group IIb subgroup, maintained up-regulated expression after Cd^2+^ stress and its homologous gene *GmWRKY142* in soybean, was reported to have enhanced Cd^2+^ resistance by up-regulating Cd^2+^ tolerance 1-Like genes and directly targeted *ATCDT1*, *GmCDT1-1*, and *GmCDT1-2* to reduce Cd^2+^ uptake (Cai et al., 2020). Nevertheless, whether *BnGWRKY46* possesses the same mechanism need to be investigated. Recent study by Dang et al. (2019) elucidated a crosstalk mechanism of *CaWRKY41*, a group III WRKY transcription factor, in immunity against the pathogen Cyanobacteria and Cd^2+^ stress response in pepper. In our study, the expression of *BnGWRKY40*, the homolog of *CaWRKY41*, rose and then fell under Cd^2+^ stress, but overall tended to be up-regulated. For this reason, we inferred that *BnGWRKY40* also has a similar function.

**FIGURE 12:**
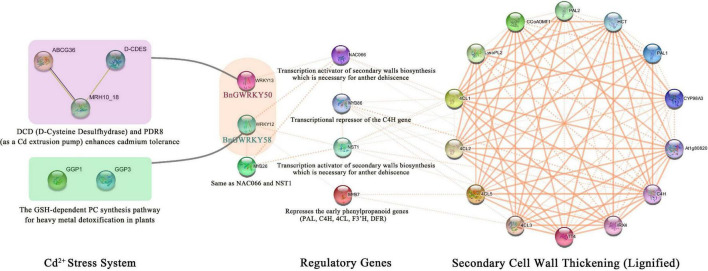
A BnGWRKY protein interaction network based on *Arabidopsis* homologs.

Protein interactions provide an intuitive and rapid understanding of gene function, especially for family genes, and are also important for the regulatory network relationships between family proteins. BnGWRKY50 and BnGWRKY58 which are homologs of AtWRKY12 and AtWRKY13 respectively were significantly activated under Cd^2+^ stress and showed a gradually increasing expression pattern from the second to the fourth stage of phloem development in the transcriptome data, which suggest multiple functions the genes. We therefore used STRING software to map a comprehensive protein interaction network based on the two *Arabidopsis* homologs (AtWRKY12 and AtWRKY13). The result (Figure 12) showed that AtWRKY12 and AtWRKY13 can mitigate the damage of Cd^2+^ stress by interacting with some proteins, such as DCD (D-Cysteine Desulfhydrase) (Zhang et al., 2020), PDR8 and GSH (Han et al., 2019) on one hand. On the other hand, they indirectly regulate the expression of key genes of the lignin metabolism pathway by binding to other transcription factors, such as NAC, MYB, and NST, which would affect the thickening of secondary cell walls, including the anther endosperm cell wall (Mitsuda et al., 2005; Wang H. et al., 2010; Li et al., 2015). This connects two disparate regulatory processes. Studies have shown that lignin biosynthesis tends to occur in specific parts of the plant, but is strongly induced when subjected to abiotic or biotic stresses (Yoon et al., 2015). This implies that BnGWRKY50 and BnGWRKY58 also have the same functions: when subjected to Cd^2+^ stress, they can both interact with proteins that alleviate the stress. At the same time, they can improve plant resistance to adversity and accelerate anther maturation and dissemination to ensure offspring reproduction through lignification (Mitsuda et al., 2005; Guillaumie et al., 2010; Wang and Dixon, 2012). Thus, this forms a crosstalk mechanism between secondary cell wall thickening and Cd2 + stress. In summary, plant resistance to natural adverse environments was an integrated regulatory network, and the Cd^2+^ stress capacity of ramie was also the result of the combined action of various mechanisms. The discovery of this crosstalk mechanism will improve our understanding of how ramie copes with stress in adversity.

## Conclusion

The WRKY transcription factors play significant role in plant metabolism and development in addition abiotic and biotic stresses responses which have been the recent research focus from the current environmental threat of climate change (Eulgem et al., 2000; Abubakar et al., 2022). It is reported to also involved in carbohydrate synthesis, senescence, development and secondary metabolite synthesis (Eulgem et al., 2000). There is no previous report to our knowledge on WRKY family in ramie. In this study, we performed a genome-wide analysis of BnGWRKYs based on which we obtained a total of 60 non-redundant BnGWRKY family members and classified into seven groups following phylogenetic and conserved structural domain analysis as group I, group II (a, b, c, d, e), and group III. Collinearity analysis showed 41.7% replication events as the main driver of BnGWRKY evolution. BnGWRKY gene promoter *cis*-acting elements and target gene predictions indicated that WRKY family members actively respond to stress stimuli such as pathogens, drought, salinity, and cold. The expression pattern of BnGWRKY genes in different tissues indicated that BnGWRKY gene played an important role in the growth and development of ramie. Transcriptome data, protein interaction network and qPCR analysis suggested a potential crosstalk mechanism between secondary cell wall thickening and Cd^2+^ stress. In addition, our preliminary screening of BnGWRKY associated with Cd^2+^ stress provides valuable information for genetic improvement of ramie tolerance to Cd^2+^ stress.

## Materials and Methods

### Identification and Analysis of BnGWRKY Family Members

To identify WRKY proteins encoded in the ramie genome, 72 *Arabidopsis* WRKY protein sequences were retrieved from TAIR^1^ and BLAST against the *B. nivea* genome. The obtained sequences were then confirmed with the aid of NCBI, CDD,^2^ and Pfam^3^ using BLAST GUI Wrapper set to default parameters in TBtools (Chen et al., 2020a). Some genes containing incomplete conserved domains were corrected using FGENESH-M. Subsequently, sequences with higher E-value were selected and aligned against SwissProt Database in NCBI. Physical and chemical properties of the BnGWRKY including length of sequences, molecular weights, hydropathicity (GRAVY) index values and theoretical isoelectric point (pI) were obtained using Protparam online analytical tool.^4^ The pLoc-mPlant tools^5^ were used to predict the subcellular localization.

### Multiple Sequence Alignment, Phylogenetic Relationship, and Classification of BnGWRKY Proteins

Multiple sequence alignments of the WRKY domains (60 *B. nivea* and 7 *A. thaliana*) based on conserved domain sequences and total [60 *B. nivea* and 72 *A. thaliana* (Wu et al., 2005)] on the basis of whole gene sequences were performed using MEGA-X with default parameters (Kumar et al., 2018). The deduced amino acid sequences in WRKY motifs were adjusted manually with Jalview software. Neighbor-joining (Guo et al., 2019) phylogenetic tree was constructed also using the MEGA-X software with the following parameters: Poisson model; pairwise deletion; and 1,000 bootstrap replications (Kumar et al., 2018). FigTree software was used to display the result.

### Structural Classification and Motif Analysis of the BnGWRKYs

The WRKYs conserve domains were obtained using the NCBI CDD. The BnGWRKYs conserved motifs were predicted using Multiple Em for Motif Elicitation (de Pater et al., 1996; Bailey et al., 2009), with optimized parameters: zero or one per sequence; the maximum number of motifs, 20; minimum width and maximum width of motifs, 6 and 100. The exon-intron organizations of the BnGWRKYs involving their distribution pattern, phases, boundaries and highlighted regions of the WRKY domains, were graphically displayed using TBtools (Chen et al., 2020a).

### BnGWRKYs Chromosome Distribution, Gene Duplication, and Synteny Analysis

BnGWRKY chromosome distribution information was obtained from the ramie genome annotation documents and genes location established with the aid of Circos (Krzywinski et al., 2009). Multiple Collinearity Scantoolkit (Wang et al., 2012) (MCscanX) was used for tandem, segmental duplication and collinearity within the species and between ramie and other species (*A. thaliana*, *C. sativa*, *Oryza sativa*, *Z. mays*) with the default parameters. The results were visualized by Advanced Circos and Multiple Synteny Plot plugin (TBtools) (Chen et al., 2020a). Synonymous (Fichman et al., 2020) and non-synonymous (Ka) substitutions of the duplicated WRKYs were obtained using KaKs_calculator 2.0 (Wang D. et al., 2010).

### *Cis*-Element Analysis

Sequences 2.0 kb upstream of each of the 60 WRKY genes were selected as the promoter region. The cis-elements in promoter sequence of BnGWRKY genes were obtained using PlantCare.^6^ All the elements were classified into four types: light-responsiveness, plant growth, stress-responsiveness and phytohormone-responsiveness, and their numbers and positions were statistically analyzed.

### Gene Ontology Functional Annotation and KEGG Enrichment Analysis of BnGWRKY Target Genes

The 2 kb DNA sequence upstream of the ATG start codon of each gene assembled from the *B. nivea* genome was used to scan the WRKY TF binding site element (W-box) with the sequence TTGAC (C/T). The BnGWRKY genes with at least four WRKY binding sites were identified as potential BnGWRKY target genes, and were used for further pathway enrichment analysis using the KEGG database (Kanehisa et al., 2017). Gene ontology (GO) annotations of the target genes were obtained using protein blast in Blast2GO tool with the default parameters (Mi et al., 2019). The output of this program was categorized into molecular function, biological processes and cellular components. The top 20 enrichment KEGG pathways and GO enrichment analysis were drawn with R package ggplot2.

### Expression Profiles of BnGWRKY Genes

The fragments per kilobase of transcript per million mapped reads (FPKM) values representing the expression levels of BnGWRKYs were computed from the *B. nivea* transcriptome data. We obtained transcriptome expression data for seven samples in NCBI SRA database, involving different tissues and different developmental stages. Detailed FPKM values were listed in Supplementary Table 7. Heat map was plotted with TBtools (Chen et al., 2020a).

### BnGWRKY Protein-Protein Interaction Network Prediction

Homology of eight selected cadmium-responsive WRKY-regulated genes between *Arabidopsis* and ramie was determined using OrthoVeen2 (Xu et al., 2019). Interaction predictions of BnGWRKY proteins with other proteins based on *Arabidopsis* homologs were obtained using the online program STRING version 11.5^7^ with a high confidence >0.400 and used to construct correlation networks (Szklarczyk et al., 2017). Interaction networks were visualized in Cytoscape v3.8.2.

### Stress Treatment (Cd^+2^) of Ramie Under Hydroponic Conditions

ZZ_1 (Zhongzhu No. 1) ramie variety was used for cadmium stress treatment following the method of Gao (Gao et al., 2018), and similar branches were selected for hydroponic cuttings. These seedlings were monitored for 15 days and those with inconsistent growth were removed. A final concentration of 50 mg/L CdCl_2_ was added and three independent biological replicates of tissues samples were collected at 0, 12, 24, and 48 h after treatment with the 0-h serving as control. All samples collected were rapidly frozen in liquid nitrogen and stored at −80°C until used.

### RNA Extraction and Expression Validation

Total RNA was extracted using SteadyPure Plant RNA Extraction Kit [Accurate Biotechnology (Changsha, Hunan) Co., Ltd.]. The RNA was reverse-transcribed (Evo M-MLV One Step RT-PCR Kit) into cDNA and quantitative RT-PCR (qPCR) analysis performed using gene-specific primers (Supplementary Table 8). 18s gene was used as an internal control (Accession Number: EU747115). The qPCR was conducted using SYBR^®^ Green Premix Pro Taq HS qPCR Kit II (Accurate Biotechnology (Changsha, Hunan) Co., Ltd.) on CFX96 Touch Deep Well Real-Time PCR System (Bio-Rad) according to standard procedure. Relative transcript levels were calculated using the 2^–ΔΔCt^ formula and the results displayed using histograms drawn with GraphPad Prism 8 software. All histograms were merged using Adobe Photoshop (2020) software.

## Data Availability Statement

The original contributions presented in the study are included in the article/Supplementary Material, further inquiries can be directed to the corresponding author.

## Ethics Statement

The plant materials were obtained from the Institute of bast fiber crops, Chinese Academy of Agricultural Sciences. Sampling of plant materials were performed in compliance with institutional, national, and international guidelines. The materials were publicly available for non-commercial purposes. No specific permits were required.

## Author Contributions

PC and XF conceived and designed the experiments. XF, AZ, XW, PM, and DS performed the experiments. XF, CY, PM, and GG analyzed the data. XF, AA, and PC wrote the manuscript. All authors have read and approved the manuscript.

## Conflict of Interest

The authors declare that the research was conducted in the absence of any commercial or financial relationships that could be construed as a potential conflict of interest.

## Publisher’s Note

All claims expressed in this article are solely those of the authors and do not necessarily represent those of their affiliated organizations, or those of the publisher, the editors and the reviewers. Any product that may be evaluated in this article, or claim that may be made by its manufacturer, is not guaranteed or endorsed by the publisher.

## Footnotes

1
https://www.arabidopsis.org/

2
https://www.ncbi.nlm.nih.gov/cdd/

3
http://pfam.xfam.org/

4
http://www.expasy.ch/tools/protparam.html

5
http://www.csbio.sjtu.edu.cn/bioinf/plant-multi/

6
http://bioinformatics.psb.ugent.be/webtools/plantcare/html/

7
https://string-db.org/

## Abbreviations

TFs: transcription factors
HTH: helix–turn–helix
MEME: multiple em for motif elicitation
NJ: neighbor–joining
ORF: open reading frame
GSDS: gene structure display server
PlantTFDB: plant transcription factor database
RNA-seq: RNA-sequencing
SMART: simple modular architecture research tool
TAIR: the *Arabidopsis* information resource
BnGWRKY: *Boehmeria nivea* WRKY gene
AtWRKY: *Arabidopsis thaliana* WRKY gene
qRT-PCR: quantitative RT-PCR
ZZ_1: Zhongzhu No.1 variety
GRAVY: grand average of hydropathicity
RNA-seq: RNA-sequencing
BLAST: basic local alignment search tool
CDS: coding sequence.
