# Supplementary figures and images for: Analysis of WRKY Resistance Gene Family in Boehmeria nivea (L.) Gaudich: Crosstalk Mechanisms of Secondary Cell Wall Thickening and Cadmium Stress
Source: Front Plant Sci. 2022 Mar 28;13:812988. doi: 10.3389/fpls.2022.812988 (PMC9010656; doi:10.3389/fpls.2022.812988)

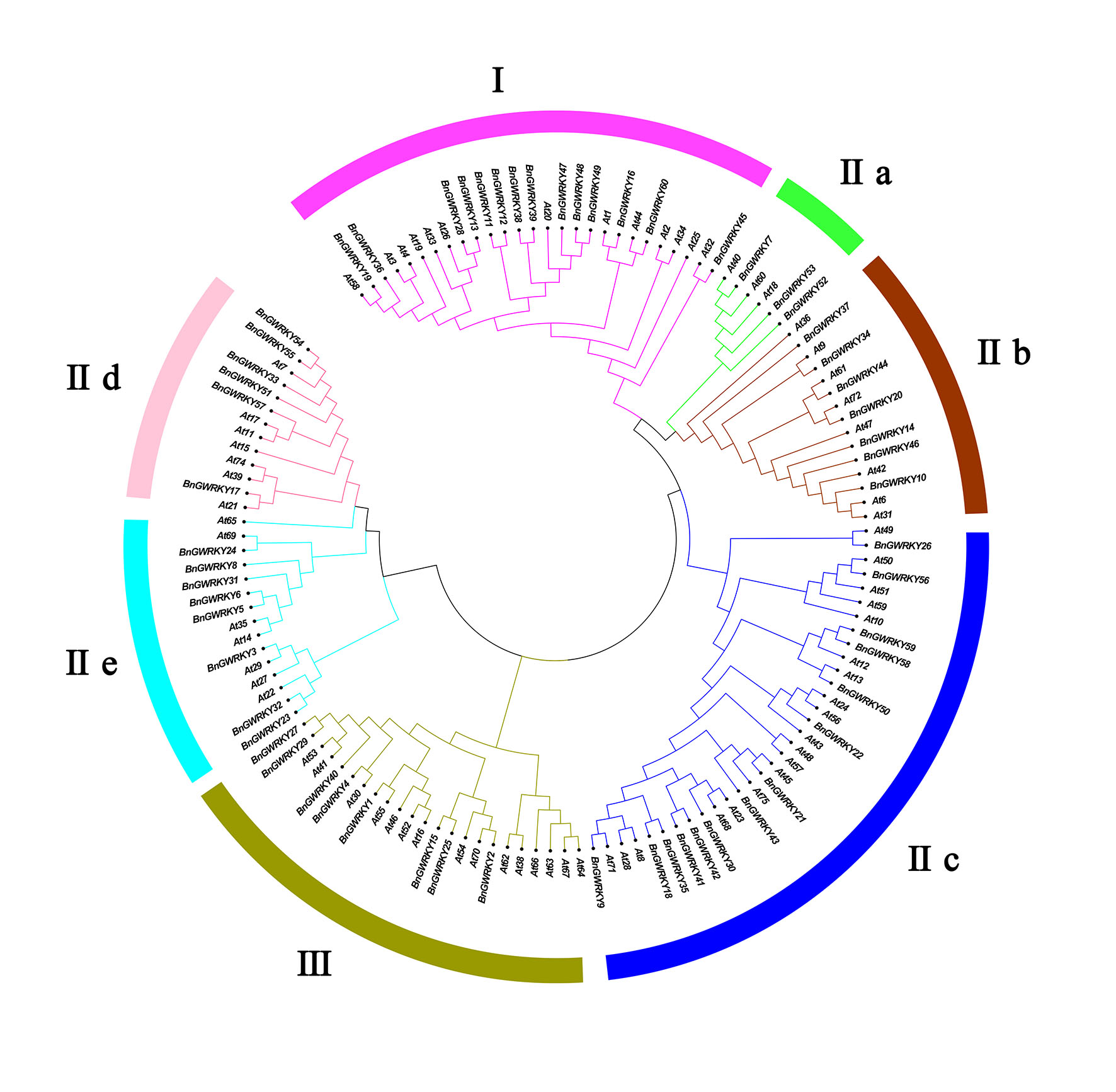

Supplement: Supplementary file 1 [file Image_1.JPEG]
