# Supplementary material for: Analysis of WRKY Resistance Gene Family in Boehmeria nivea (L.) Gaudich: Crosstalk Mechanisms of Secondary Cell Wall Thickening and Cadmium Stress
Source: Front Plant Sci. 2022 Mar 28;13:812988. doi: 10.3389/fpls.2022.812988 (PMC9010656; doi:10.3389/fpls.2022.812988)
Supplement: Supplementary file 2 [file Table_9.pdf]

Figure S2: The directed acyclic graph for the enriched terms based on GO.

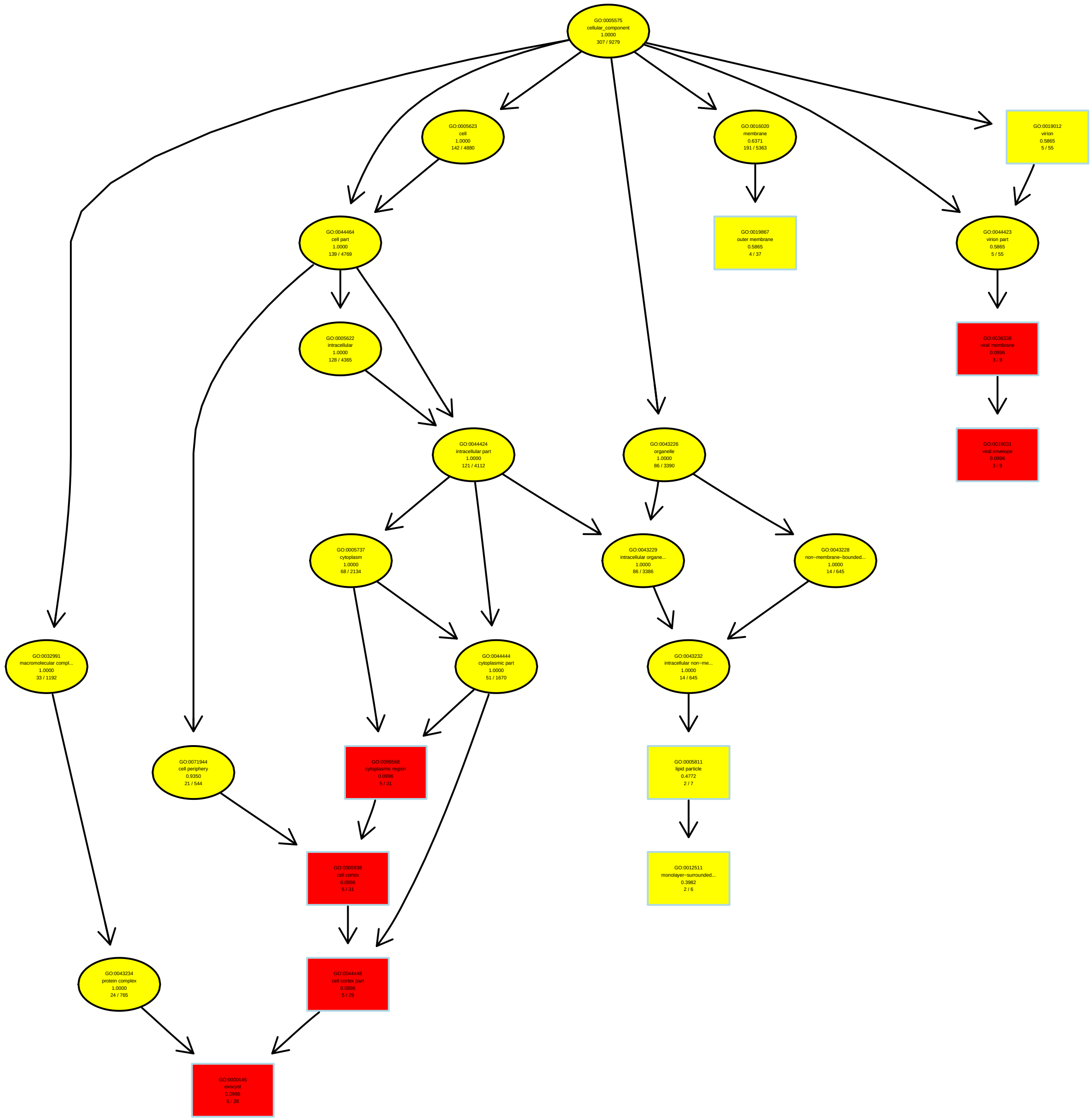

A: Cellular Component

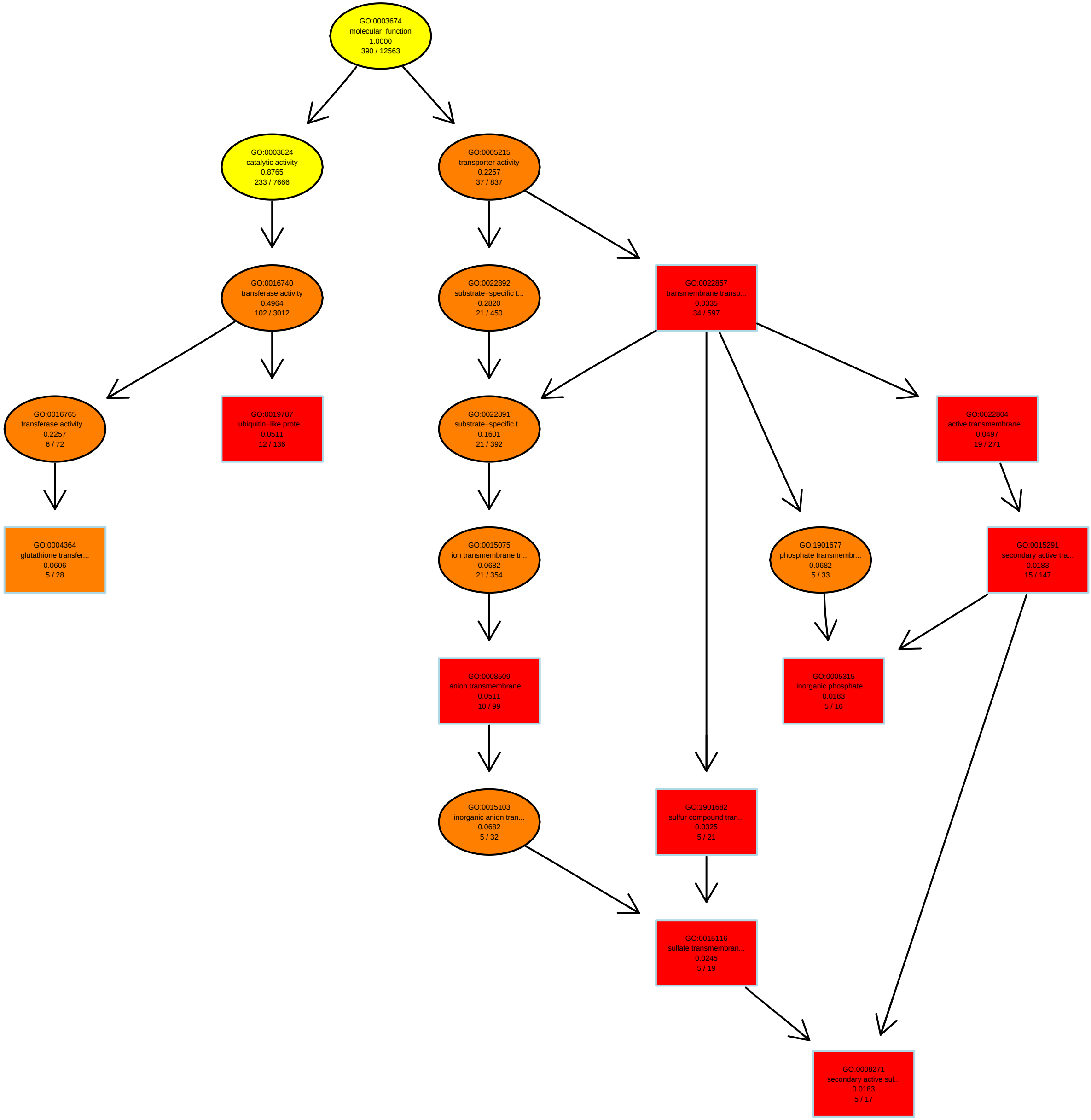

B: Molecular Function

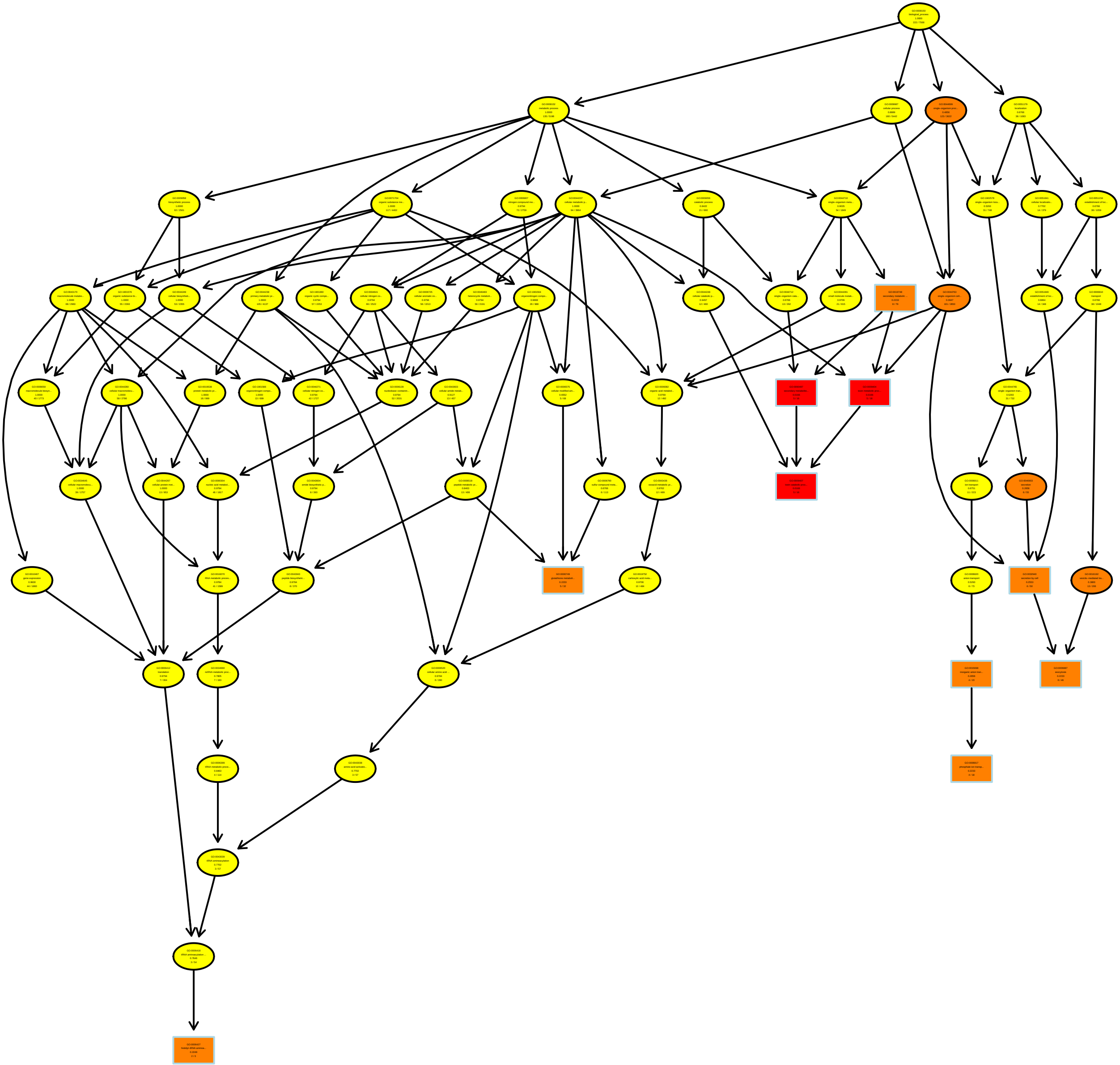

C: Biological Process
